# Myocardial triglycerides in cardiac amyloidosis assessed by proton cardiovascular magnetic resonance spectroscopy

**DOI:** 10.1186/s12968-019-0519-6

**Published:** 2019-01-31

**Authors:** Mareike Gastl, Sophie M. Peereboom, Alexander Gotschy, Maximilian Fuetterer, Constantin von Deuster, Florian Boenner, Malte Kelm, Rahel Schwotzer, Andreas J. Flammer, Robert Manka, Sebastian Kozerke

**Affiliations:** 1 Institute for Biomedical Engineering, University and ETH Zurich, Gloriastrasse 35, 8092 Zurich, Switzerland; 2 Department of Cardiology, University Heart Center, University Hospital Zurich, Zurich, Switzerland; 3 Department Cardiology, Pneumology and Angiology, Heinrich Heine University, Düsseldorf, Germany; 4 Comprehensive Cancer Center Zürich, University Hospital Zurich, Zurich, Switzerland; 5 Institute of Diagnostic and Interventional Radiology, University Hospital Zurich, Zurich, Switzerland

**Keywords:** Cardiac amyloidosis, Cardiovascular magnetic resonance, Proton spectroscopy, Myocardial metabolism, Left-ventricular thickening

## Abstract

**Background:**

Cardiac involvement of amyloidosis leads to left-ventricular (LV) wall thickening with progressive heart failure requiring rehospitalization. Cardiovascular magnetic resonance (CMR) is a valuable tool to non-invasively assess myocardial thickening as well as structural changes. Proton CMR spectroscopy (^1^H-CMRS) additionally allows assessing metabolites including triglycerides (TG) and total creatine (CR). However, opposing results exist regarding utilization of these metabolites in LV hypertrophy or thickening. Therefore, the aim of this study was to measure metabolic alterations using ^1^H-CMRS in a group of patients with thickened myocardium caused by cardiac amyloidosis.

**Methods:**

^1^H-CMRS was performed on a 1.5 T system (Achieva, Philips Healthcare, Best, The Netherlands) using a 5-channel receive coil in 11 patients with cardiac amyloidosis (60.5 ± 11.4 years, 8 males) and 11 age- and gender-matched controls (63.2 ± 8.9 years, 8 males). After cardiac morphology and function assessment, proton spectra from the interventricular septum (IVS) were acquired using a double-triggered PRESS sequence. Post-processing was performed using a customized reconstruction pipeline based on ReconFrame (GyroTools LLC, Zurich, Switzerland). Spectra were fitted in jMRUI/AMARES and the ratios of triglyceride-to-water (TG/W) and total creatine-to-water (CR/W) were calculated.

**Results:**

Besides an increased LV mass and a thickened IVS concomitant to the disease characteristics, patients with cardiac amyloidosis presented with decreased global longitudinal (GLS) and circumferential (GCS) strain. LV ejection fraction was preserved relative to controls (60.0 ± 13.2 vs. 66.1 ± 4.3%, *p* = 0.17). Myocardial TG/W ratios were significantly decreased compared to controls (0.53 ± 0.23 vs. 0.80 ± 0.26%, *p* = 0.015). CR/W ratios did not show a difference between both groups, but a higher standard deviation in patients with cardiac amyloidosis was observed. Pearson correlation revealed a negative association between elevated LV mass and TG/W (*R* = − 0.59, *p* = 0.004) as well as GCS (*R* = − 0.48, *p* = 0.025).

**Conclusions:**

A decrease in myocardial TG/W can be detected in patients with cardiac amyloidosis alongside impaired cardiac function with an association to the degree of myocardial thickening. Accordingly, ^1^H-CMRS may provide an additional diagnostic tool to gauge progression of cardiac amyloidosis along with standard imaging sequences.

**Trial registration:**

EK 2013–0132.

## Background

Amyloidosis is a multisystemic disorder which is characterized by extracellular deposition of misfolded amyloid proteins leading to consecutive organ failure [1]. Cardiac involvement is frequent. The most common types are acquired or inherited transthyretin-related (ATTR) and light-chain (AL) amyloidosis. Once affected, prognosis and outcome of patients with cardiac amyloidosis deteriorates dependent on the subtype, hence requiring sufficient diagnostic accuracy and follow-up strategies [2, 3]. Currently, definite diagnosis of cardiac amyloidosis is established using either invasive tissue biopsy for all types or technetium scintigraphy in ATTR amyloidosis respectively [4, 5]. Cardiovascular magnetic resonance (CMR) is increasingly used for screening purposes in patients with suspected cardiac involvement of amyloidosis due to its non-invasive nature and its potential to characterize different forms of amyloidosis [6, 7]. Typical cardiac manifestations include myocardial thickening and diffuse late gadolinium enhancement (LGE) resulting from expanded extracellular space. However, the expression of cardiac amyloidosis and its sub-forms can be heterogeneous and the differentiation from other forms of myocardial thickening may be challenging at early stages [1]. Besides the detection of morphological and structural changes with CMR, proton spectroscopy may offer additional insights into the metabolism of healthy and diseased hearts [8, 9]. In particular, myocardial triglyceride (TG) and total creatine (CR) content can be determined [9].

In the healthy human heart, β-oxidation of fatty acids constitutes the major mechanism of energy production that is subject to storage of TG [10]. In thickened hearts, mainly in left-ventricular (LV) hypertrophy (LVH), studies have provided controversial results about TG utilization. In particular, myocardial TG was found to be significantly reduced in hypertrophic cardiomyopathy (HCM) while no changes were found in LVH associated with Anderson-Fabry’s disease [11–13]. Potential metabolic alterations involved in myocardial thickening due to cardiac amyloidosis have not been studied in detail and previous histological and proton spectroscopy studies present with contradictory results. On the one hand, reduced TG utilization has been found to associate with advanced stages of heart failure and HCM while, on the other hand, histopathological analyses revealed an association of fatty acids with amyloid proteins [8, 14, 15].

Therefore, the objective of the present study was to assess myocardial TG and additional CR content along with functional parameters in a group of patients with cardiac amyloidosis using CMR imaging and spectroscopy.

## Methods

The study was conducted in accordance to the declaration of Helsinki and its later amendments. Local ethics committee approval was obtained. All data acquired in this study were handled anonymously and written informed consent was obtained from each subject.

### Study population

Eleven patients with cardiac amyloidosis (60.5 ± 11.4 years, 8 males, AL = 7, ATTR = 4) were recruited from the interdisciplinary “Amyloidosis-Network” of the University Hospital Zurich. Cardiac involvement of systemic amyloidosis was confirmed by endomyocardial biopsy, biopsy of other tissue (fat, lips) or by imaging [4, 16]. Amyloidosis patients as well as 11 age- and gender matched controls (63.2 ± 8.9 years, 8 males) received CMR imaging and spectroscopy at the University Hospital Zurich, Switzerland. In addition, blood lipid- and N-terminal prohormone of brain natriuretic peptide-levels (NT-proBNP) were measured by acquiring blood samples. Subjects were advised to adhere to a 3-h fasting period prior to the CMR examination and scans were performed at afternoon times.

### Data acquisition

CMR imaging and spectroscopy was performed on a 1.5 T system (Achieva, Philips Healthcare, Best, The Netherlands) using a 5-channel phased array coil. After scout and reference scans, anatomical and geometric data were acquired using balanced steady-state free precession (bSSFP) sequence in standard long-axis geometries (two-, three- and four-chamber view) as well as in short-axis view with full LV coverage from base to apex (repetition time (TR)/echo time (TE) = 3.3 / 1.6 ms, flip angle = 60°, spatial resolution = 1.5 × 1.5 × 8 mm^3^, 50 phases, 2 slices per breathhold).

In cases with an estimated glomerular filtration rate (eGFR) > 35 mL/min and informed consent of the subject, a gadolinium-based contrast agent (Gadovist, Bayer Healthcare, Berlin, Germany) was injected for LGE imaging to visualize fibrosis and scarring. Ethical approval constrains only allowed the administration of contrast agent in amyloidosis patients. All patients were eligible to receive gadolinium. After a period of 10 min, a 3-dimensional gradient spoiled turbo fast-field echo sequence with a non-selective 180° inversion pre-pulse was performed at end diastole (TR/TE = 3.3/1.6 ms, flip angle = 15°, spatial resolution = 1.6 × 1.6 × 5 mm^3^, adjusted inversion delay, one breath-hold for each anatomical location) in the same anatomical location as the bSSFP scans [17].

### Spectroscopy

**Fig. 1:**
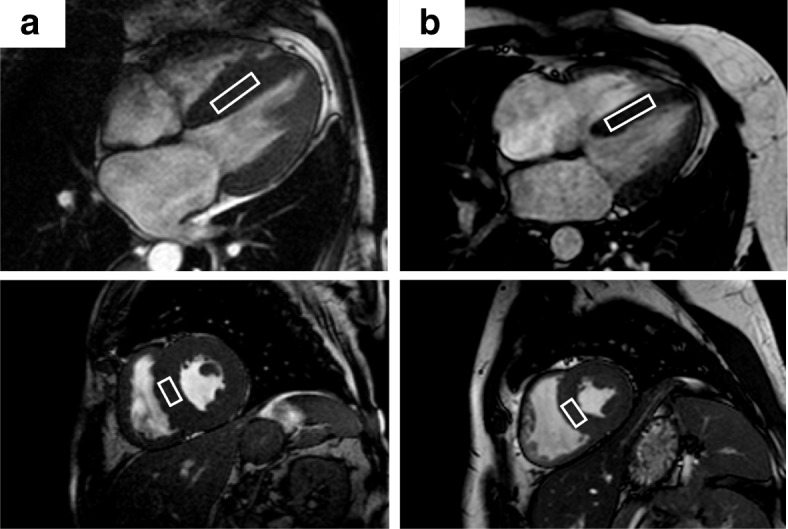
Position of the ^1^H-CMRS voxel within the IVS of a patient with cardiac amyloidosis (**a**) and of a normal control (**b**). IVS, interventricular septum; ^1^H-CMRS, Proton CMR spectroscopy

Spectroscopy data were acquired after anatomical cine imaging and prior to contrast agent injection. Cine imaging in four-chamber and short-axis views were repeated with navigator gating on the diaphragm for subsequent planning of the spectroscopic voxel and to account for potential subject movement during the scan session. Prior to the acquisition of spectroscopy data, iterative volume shimming was performed over a volume of 15 × 25 × 45 mm^3^ during a single breathhold. Thereafter, voxels of 7–8 mL (10 × 20 × 35–40 mm^3^) were positioned in the interventricular septum (IVS) and proton spectra were recorded using a PRESS (point-resolved spectroscopy) sequence in systole with optimized spoilers (Fig. 1) [18]. Spectroscopic data acquisition was double-triggered using electrocardiogram (ECG) triggering and pencil beam navigator-based respiratory gating on the diaphragm with a gating window of 4 mm. Water suppression was achieved by chemical shift selective saturation (CHESS) (excitation bandwidth 100 Hz) [19]. A total of 96 averages with water suppression and 16 averages without water suppression were recorded at a spectral bandwidth of 2000 Hz (1024 samples) and a minimum TR of 2000 ms (TE = 22 ms, flip angle = 90°).

### Post-processing

Functional and geometric parameter assessment based on imaging data was achieved using commercial post-processing software (IntelliSpace Portal, Philips, Best, The Netherlands). The bSSFP imaging data were used to measure the IVS at maximum extension in end diastole, LV and right ventricular (RV) end-diastolic volume (LVEDV/RVEDV), left ventricular end-diastolic diameter (LVEDD), left and right ventricular ejection fraction (LVEF/RVEF), stroke volume (SV), and LVM indexed to body surface area (LVMi).

LV strain and strain rate analysis was performed using feature tracking analysis (Image-Arena VA Version 4.6 and 2D Cardiac Performance Analysis MR Version 4, TomTec Imaging Systems, Unterschleissheim, Germany). The bSSFP images were used to manually draw endocardial contours followed by software-driven automatic tracking. End-systolic global longitudinal (GLS) and circumferential strain (GCS) as well as peak diastolic longitudinal and circumferential strain rate were extracted.

**Fig. 2:**
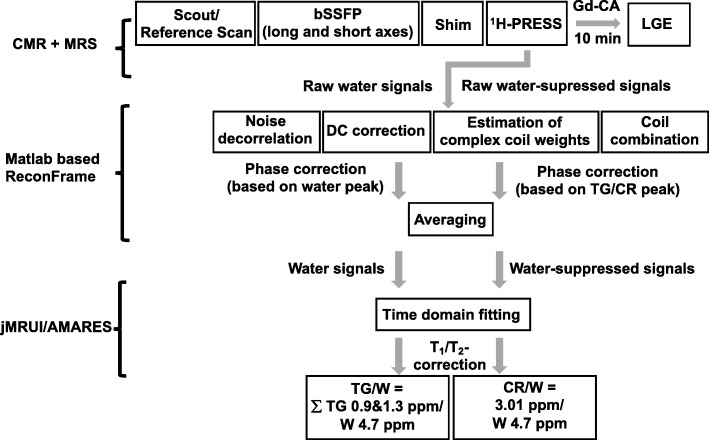
Flow chart of CMR imaging and spectroscopy as well as processing pipeline for spectroscopy data. *CR,* Creatine; *Gd-CA,* gadolinium based contrast agent; LGE, late gadolinium enhancement; *CMRS*, cardiovascular magnetic resonance spectroscopy; bSSFP*,* balanced steady state free precession; *PRESS,* point-resolved spectroscopy; *TG,* triglycerides

Spectroscopy data were first reconstructed in MATLAB (The Mathworks, Natick, Massachusetts, USA) using a customized reconstruction pipeline based on ReconFrame (GyroTools LLC, Zurich, Switzerland) as illustrated in Fig. 2*.* After noise decorrelation, a singular value decomposition approach (SVD) was used for the combination of all five individual coil channels. Weights estimated from the water spectra were used for the calculation of water-unsuppressed and water-suppressed signals [20, 21]. Following this step, individual averages were phase-corrected. For non-water suppressed spectra, phasing was based on the water peak whereas for water-suppressed spectra, phasing was based on the peak at 1.3 ppm for TG and on the peak at 3.01 ppm for CR. Afterwards, averaging was conducted.

Reconstructed and averaged spectra were analysed in jMRUI/AMARES (version 5.2) [22] Water-unsuppressed and water-suppressed spectra were time-domain fitted using Lorentzian line shapes after first-order phase correction. A Hankel-Lanczos Singular Value Decomposition (HLSVD) approach was used to filter out the residual water peak and a total of six resonances were fitted to the water-suppressed spectra: (1) TG at 0.9, 1.3 and 2.1 ppm, (2) CR at 3.01 ppm, (3) trimethylammonium (TMA) at 3.2 ppm and (4) the residual water at 4.7 ppm. The maximum linewidth of all resonances was limited by soft constraints to 0.75 ppm and relative chemical shifts were used as fixed boundary conditions.

For the calculation of triglyceride- and creatine-to-water ratios (TG/W and CR/W), data were first corrected for longitudinal and transverse relaxation by using T_1_ = 970 ms and T_2_ = 40 ms for myocardial water, T_1_ = 280 ms and T_2_ = 80 ms for myocardial TG, and T_1_ = 1480 ms and T_2_ = 135 ms for CR [21, 23]. The TG/W ratio was then calculated as the sum of the fitted TG resonances at 0.9 and 1.3 ppm divided by the fitted water. The CR/W ratio was calculated from the CR resonance at 3.01 ppm divided by the fitted water signal.

The amount of fibrosis as percentage of LVMi was calculated semi-automatically by manually applying epi- and endocardial contours to the short-axis LGE images for every slice. Then, the amount of fibrosis was calculated using a full-width-at-half-maximum (FWHM) approach as previously described [24]**.**

### Statistical analysis

Statistical analyses were conducted using SPSS (version 24.0, International Business Machines, Armonk, New York, US). Continuous variables are expressed as mean ± standard deviation (SD). Categorical variables are reported as percentages. After testing for normal distribution using a Kolmogorov-Smirnov test, data between groups were compared using a 2-sided unpaired Student’s t-test for normally distributed data and a non-parametric Mann-Whitney *U*-tests for not normally distributed data. Fisher’s exact t-test was used to examine significant differences between nominal classifications. Bivariate Pearson correlation analysis was conducted to analyze relationships between different CMR parameter including ^1^H-CMRS results. *P*-values below 0.05 were considered as statistically significant.

## Results

### Study population and clinical characteristics

Cardiovascular comorbidities, age- and sex were not significantly different between amyloidosis patients and the control group. Biochemical analyses revealed no differences in blood lipid levels between the groups (Table 1). NT-proBNP of amyloidosis patients was elevated according to recent heart failure guidelines [25].

**Table 1:** Demographic and clinical baseline characteristics

	Amyloidosis (*n* = 11)	Controls (*n* = 11)	*P*-value
Age (years)	60.5 ± 11.4	62.9 ± 8.9	0.57
Male (%)	8 (73)	8 (73)	1.0
BMI (g/m^2^)	24.3 ± 2.9	24.2 ± 4.6	0.94
AL-Amyloidosis	7 (64)	–	–
Disease duration (years)	2.19 ± 2.1	–	–
Comorbidities			
Diabetes, *n*(%)	1 (9)	0 (0)	0.31
Hypertension, *n*(%)	4 (36)	5 (45)	0.66
CAD, n(%)	0 (0)	0 (0)	1.0
Previous stroke, *n*(%)	1 (9)	0 (0)	0.31
Class NYHA III-IV *n*(%)	2 (18)	0 (0)	0.14
Biochemical			
Cholesterol (mmol/L)	5.9 ± 3.2	5.6 ± 1.0	0.794
HDL (mmol/L)	1.3 ± 0.5	1.5 ± 0.4	0.187
Non-HDL (mmol/L)	4.5 ± 3.4	4.0 ± 1.0	0.664
LDL (mmol/L)	2.7 ± 1.2	3.3 ± 0.8	0.234
Triglycerides (mmol/L)	2.5 ± 2.9	1.7 ± 0.9	0.358
NT-proBNP (ng/L)	1287 ± 1304	–	–
Creatinine (μg/dL)	110 ± 33.9	–	–
Treatment			
Stem cell transplantation	4 (36)	–	–
Chemotherapy	7 (64)	–	–
Green tea	3 (27)	–	–

*AL* amyloid light-chain, *BMI* body mass index, *CAD* coronary artery disease, *GFR* glomerular filtration rate, *HDL* high density lipoprotein, *LDL* low density lipoprotein, *NYHA* New York Heart Association, *NT-proBNP* N-terminal prohormone of brain natriuretic peptide-levels

### Imaging

Baseline CMR parameters of amyloidosis patients and controls are summarized in Table 2. Amyloidosis patients showed significantly elevated IVS and LVMi. LVEF as one surrogate for myocardial systolic function did not show a significant alteration, but GLS and GCS were significantly reduced compared to controls (*p* < 0.001 for GLS and *p* = 0.007 for GCS). Circumferential peak longitudinal strain rate as a surrogate for diastolic function was decreased in amyloidosis patients (*p* = 0.019). Fibrosis extent in the amyloidosis patients ranged from 2 to 74% (mean: 36.1 ± 27.1%) of the LV wall.

**Table 2:** Baseline CMR characteristics of patients and controls

	Amyloidosis	Controls	*P-value*
LVEF (%)	60.0 ± 13.2	66.1 ± 4.3	0.170
IVS (mm)	18.0 ± 4.6	8.0 ± 1.5	< 0.001
LVMi(g/m^2^)	85.2 ± 25.1	45.0 ± 8.7	< 0.001
LVEDV (mL)	130.1 ± 23.0	126.9 ± 34.0	0.8
LVEDD (mm)	46.2 ± 3.5	46.9 ± 4.6	0.678
SV (mL)	76.7 ± 16.3	84.5 ± 20.2	0.331
RVEF (%)	64.5 ± 9.5	62.8 ± 5.0	0.604
RVEDV (mL)	112.0 ± 30.5	130.1 ± 29.3	0.171
GLS (%)	−19.7 ± 5.6	−31.1 ± 5.5	< 0.001
GCS (%)	−29.8 ± 6.1	−36.2 ± 3.3	0.007
Long SRe	1.7 ± 0.5	1.4 ± 0.4	0.076
Circ SRe	1.3 ± 0.3	1.7 ± 0.4	0.019

*Circ* circumferential, *GLS* global longitudinal strain, *GCS* global circumferential strain, *IVS* interventricular septum, *Long* longitudinal, *LVEDD* left ventricular end-diastolic diameter, *LVEDV/RVEDV* left/right ventricular end-diastolic volume, *LVEF/RVEF* left/right ventricular ejection fraction, *LVMi* left ventricular mass indexed to body surface area, *SRe* strain rate, *SV* stroke volume

Apart from an elevated LVEDV (AL vs. ATTR: 119.4 ± 20.1 vs. 148.8 ± 14.9, *p* = 0.032) and a trend towards a decrease in GLS and GCS (AL vs. ATTR: GLS = − 21.9 ± 4.5 vs. -15.8 ± 5.7%, *p* = 0.08; GCS = − 32.4 ± 5.4 vs. -25.3 ± 4.7%, *p* = 0.058) for the ATTR-type, there was no significant difference between both groups*.*

At the time of the CMR exam, 4 amyloidosis patients had already received autologous stem cell transplantation. Those patients showed improved GCS and circumferential strain rate in comparisons to the medically treated patients (GCS = − 34.5 ± 5.0 vs. -27.2 ± 5.2%, *p* = 0.048; circumferential strain rate = 1.5 ± 0.3 vs. 1.2 ± 0.2 s^− 1^, *p* = 0.06) with no statistical significance compared to the normal controls.

### Spectroscopy

**Fig. 3:**
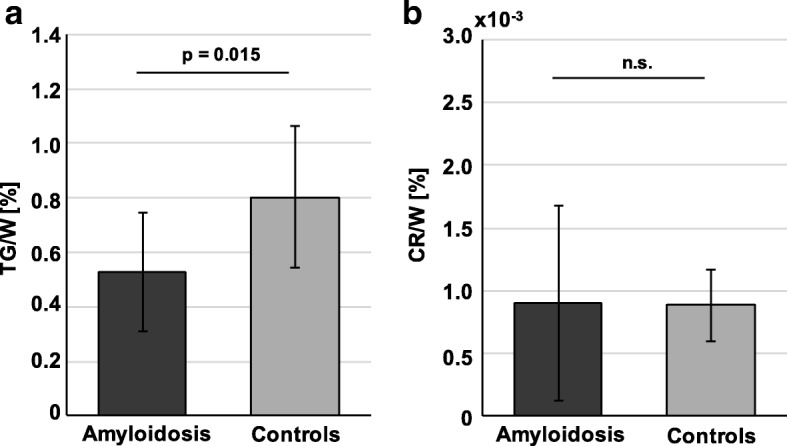
Mean and SD of TG/W and CR/W ratios in patients with amyloidosis compared to an age- and BMI-matched normal control group. TG/W, triglyceride-to-water; Cr/W, creatine-to-water; SD, standard deviation

**Fig. 4:**
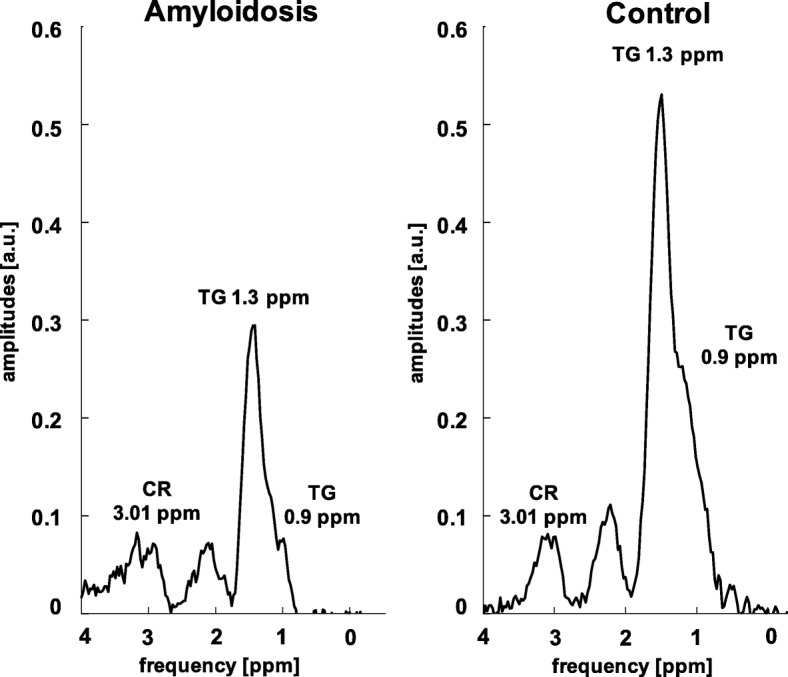
Exemplary spectra in a patient with amyloidosis and in a control. TG/W, triglyceride-to-water; CR/W, creatine-to-water; ppm, part per million

Spectroscopic data revealed decreased TG/W ratios of 0.53 ± 0.23% for the cardiac amyloidosis group in comparison to the control group (0.80 ± 0.26%, *p* = 0.015) (Figs. 3 & 4). Myocardial CR/W ratios did not show significant differences between both groups, although amyloidosis patients exhibited a larger variation by means of SD (amyloidosis vs controls: 0.09 ± 0.08 vs. 0.09 ± 0.03%). Dividing amyloidosis patients according to their type of amyloidosis or according to their previous treatment (+/− stem cell therapy), no differences in TG/W and CR/W ratios could be detected.

### Correlation analysis

**Fig. 5:**
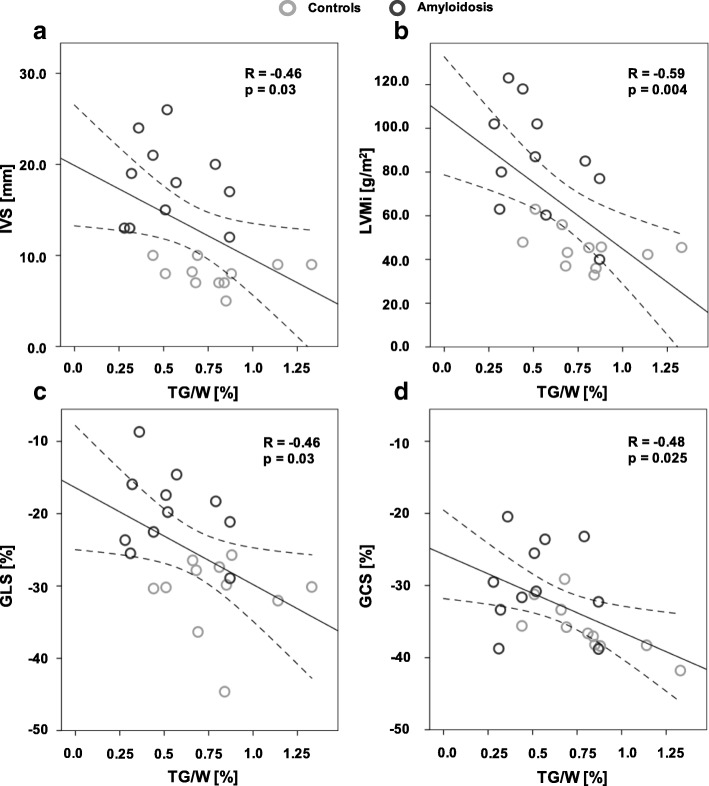
Pearson’s correlation between TG/W ratios and (**a**) IVS and (**b**) LVMi, (**c**) GLS as well as (**d**) GCS. GCS, global circumferential strain; GLS, global longitudinal strain; IVS, interventricular septum; LVMi, left-ventricular mass indexed to body surface area; TG/W, triglyceride-to-water

Figure 5 shows bivariate Pearson’s correlation between imaging parameters of LV-hypertrophy or function and TG/W ratios. Decreasing TG/W ratios with increasing LVMi and IVS were found (IVS: *R* = − 0.46, *p* = 0.033; LVMi: *R* = − 0.59, *p* = 0.004).

Comparing myocardial functional LV parameters, there was no correlation between TG/W and LVEF, but there was for TG/W and GLS (R = − 0.46, p = 0.033) and GCS (*R* = − 0.48, *p* = 0.025). Fibrosis, age, NT-proBNP and blood lipid levels did not show any correlations. CR/W did not correlate to any of those parameters.

## Discussion

In the present study, CMR spectroscopy identified reduced TG/W ratios, but no change of CR/W in patients with cardiac amyloidosis when compared to age-matched controls. TG/W was negatively correlated with the severity of LV thickening and LV strain, but not with LVEF.

The reduced myocardial TG/W ratios in the myocardium of patients with amyloidosis are in line with previous studies describing reduced TG content in patients with HCM [8, 11]. Fatty acids (FA) are the major source for myocardial energy in the healthy heart [9, 10]. However myocardial FA oxidation is impaired in heart failure, which in turn, may also trigger myocardial TG accumulation [8, 26, 27]. Recent literature and the current findings indicate that the pathophysiology of myocardial thickening and/or LVH may play an additional role in myocardial TG accumulation. In HCM, decreasing TG content has been reported [11]. In secondary LVH (e.g. valvular or Anderson-Fabry’s disease) different results can be found depending on the pathogenesis [13, 28]. In mild hypertrophy caused by competitive sports, TG content was preserved [28]. Surprisingly, Anderson-Fabry’s disease that presents with an accumulation of sphingolipids did not associate with an increased TG content [13].

In the general context, CMR spectroscopy studies suggest that accumulation of lipids in LVH and heart failure is most pronounced when associated with obesity, overweight or diabetes [29, 30]. Only one subject of the present study group suffered from diabetes. Indeed, the person with cardiac amyloidosis suffering from diabetes and additional overweight (body mass index (BMI) > 25 kg/m^2^) exhibited one of the highest TG/W ratios within the amyloidosis cohort. This indicates that these two risk factors might offset the reduction in TG/W ratios. TG/W ratios did not correlate with BMI, blood lipid levels or age in the present study, suggesting that TG/W ratios may associate with other factors, such as the accumulation of amyloid proteins. This is indicated by the negative relationship between TG content and the severity of the thickened myocardium (Fig. 5) and a known histologic correlation of amyloid and the amount of fibrosis [31].

Amyloid proteins of any type mainly deposit within the myocardial interstitium and extend the extracellular volume which may hamper extramyocellular TG deposition [32, 33]. So far, the direct influence of amyloid on TG is unknown, but an increasing effect on the T_1_-values of the global myocardium has already been described [3]. As a consequence of the prolonged T_1_-values of tissue water, partial saturation increases using PRESS with a fixed TR of 2000 ms. Correcting the water signal for those T_1_-values would increase the signal by approximately 10% and therefore the TG/W ratio would further decrease below the value stated above. Another possible mechanism may relate to the myocytotoxic effects of circulating amyloid light chains and their influence on mitochondrial function, i.e. the β-oxidation of free fatty acids [34–36]. This interaction causes oxidative stress of the cell leading to the toxic effects in proteins, lipids, DNA and inflammatory processes [27]. Not only amyloid light chains interact with free FAs, but also the amyloid of Alzheimer’s disease that reduces unsaturated fatty acids in certain parts of the brain known to cause dementia [37]. However, further research of the exact mechanism of metabolic alterations in cardiac amyloidosis has to be pursued.

Results in the age-matched controls were in line with previous literature [38, 39]. To address diurnal changes of TG/W, subjects and patients were scanned during afternoon hours and with a prior fasting period of about 3 h [40].

No mean difference of CR/W could be detected between patients and controls. However, SD of myocardial CR was higher in the amyloidosis group potentially due to different disease states of the patients [41, 42]. In contrast to previous studies, no correlation between CR/W ratios and LV function parameters could be detected [10, 43]. It should, however, be noted that fitting of CR peaks is challenging due to their low signal and hence results may be confounded by fitting errors [9].

Although ^1^H-CMRS has not yet found its way into clinical routine due to technical demands and the still time-consuming scan, ^1^H-CMRS may add clinical value by detecting metabolic changes in the course of developing cardiac amyloidosis. Considering the current progress regarding scan time reduction and ease-of-use of ^1^H-CMRS, there is the potential to integrate the method into standard imaging workflows.

Besides LGE, parametric imaging techniques including T_1_ and extracellular volume (ECV) mapping have been used in cardiac amyloidosis to distinguish different amyloidosis types [44, 45]. Both LGE and ECV mapping require gadolinium injection which excludes patients with severe renal insufficiency. In these patients, expansion of ECV may be identified through changes in the triglyceride levels using ^1^H-CMRS. Moreover, modulation of TG/W values may point directly to myocytotoxic effects of circulating amyloid chains which potentially contribute to ECV expansion as measured with T_1_/ECV mapping [27]. These insights, however, remain to be established through larger studies in the future.

### Limitations

The present sample size was relatively small due to the technical demands of the study. Subject numbers were however comparable to previous studies assessing myocardial metabolism in heart failure [11, 28]. Assuming decreased myocardial TG/W according to literature, we sought to identify an effect in comparison to normal controls with a statistical power of 90% and type I error of less than 5%, resulting in an estimated sample size of 10 per group [11].

As cardiac amyloidosis had already been confirmed in all patients at the time of CMR, we did not perform myocardial biopsies to quantify myocardial TG content and to differentiate intramyocellular (IMCL) and extramyocellular lipids (EMCL). The differentiation of IMCL and EMCL may help to understand the mechanism of the TG/W reduction in cardiac amyloidosis and whether it is caused by expanded extracellular space or by a reduced metabolism of myocytes. Although recent work has shown feasibility to quantify IMCL and EMCL in the human heart using spectroscopy, the present method could not reliably differentiate between the different pools [46].

## Conclusions

Myocardial TG/W decreases in cardiac amyloidosis and is associated with the severity of myocardial thickening and systolic dysfunction, independent of age, BMI and blood lipid levels.CMR proton spectroscopy may provide additional information to gauge progression of cardiac amyloidosis.

## Abbreviations

^1^H-CMRS: Cardiac proton magnetic resonance spectroscopy
AL: Light-chain amyloidosis
AS: Aortic stenosis
ATTR: Transthyretin-related amyloidosis
BMI: Body mass index
bSSFP: balanced steady-state free precession
CAD: Coronary artery disease
CHESS: Chemical shift selective saturation
CMR: Cardiovascular magnetic resonance
CR: Creatine
CR/W: Creatine-to-water ratio
ECG: Electrocardiogram
ECV: Extracellular volume
eGFR: Estimated glomerular filtration rate
EMCL: Extramyocellular lipids
FA: Fatty acids
FWHM: Full width at half maximum
GCS: Global circumferential strain
GLS: Global longitudinal strain
HCM: Hypertrophic cardiomyopathy
HDL: High density lipoprotein
HLSVD: Hankel-Lanczos Singular Value Decomposition
IMCL: intramyocellular lipids
IVS: Interventricular septum
LDL: Low density lipoprotein
LGE: Late gadolinium enhancement
LVEDV: Left ventricular end-diastolic volume
LVEF: Left ventricular ejection fraction
LVH: Left ventricular hypertrophy
LVM(i): Left ventricular indexed mass
NT-proBNP: N-terminal prohormone of brain natriuretic peptide
NYHA: New York Heart Association
PRESS: Point-resolved spectroscopy
RVEDV: Right ventricular end-diastolic volume
RVEF: Right-ventricular ejection fraction
SD: Standard deviation
SRe: Strain rate
SV: Stroke volume
SVD: Singular value decomposition
TE: Echo time
TG: Triglyceride
TG/W: Triglyceride-to-water ratio
TMA: Trimethylammonium
TR: Repetition time
VOI: Volume of interest
